# Non-invasive technologies for heart failure, systolic and diastolic dysfunction modeling: a scoping review

**DOI:** 10.3389/fbioe.2023.1261022

**Published:** 2023-10-18

**Authors:** Sona M. Al Younis, Leontios J. Hadjileontiadis, Cesare Stefanini, Ahsan H. Khandoker

**Affiliations:** ^1^ Department of Biomedical Engineering, Healthcare Engineering Innovation Center (HEIC), Khalifa University, Abu Dhabi, United Arab Emirates; ^2^ Creative Engineering Design Lab at the BioRobotics Institute, Applied Experimental Sciences Scuola Superiore Sant'Anna, Pontedera (Pisa), Italy

**Keywords:** heart failure, myocardial dysfunction, non-invasive sensing, systolic dysfunction, ballistocardiogram (BCG), impedance cardiography (ICG), photoplethysmography (PPG)

## Abstract

The growing global prevalence of heart failure (HF) necessitates innovative methods for early diagnosis and classification of myocardial dysfunction. In recent decades, non-invasive sensor-based technologies have significantly advanced cardiac care. These technologies ease research, aid in early detection, confirm hemodynamic parameters, and support clinical decision-making for assessing myocardial performance. This discussion explores validated enhancements, challenges, and future trends in heart failure and dysfunction modeling, all grounded in the use of non-invasive sensing technologies. This synthesis of methodologies addresses real-world complexities and predicts transformative shifts in cardiac assessment. A comprehensive search was performed across five databases, including PubMed, Web of Science, Scopus, IEEE Xplore, and Google Scholar, to find articles published between 2009 and March 2023. The aim was to identify research projects displaying excellence in quality assessment of their proposed methodologies, achieved through a comparative criteria-based rating approach. The intention was to pinpoint distinctive features that differentiate these projects from others with comparable objectives. The techniques identified for the diagnosis, classification, and characterization of heart failure, systolic and diastolic dysfunction encompass two primary categories. The first involves indirect interaction with the patient, such as ballistocardiogram (BCG), impedance cardiography (ICG), photoplethysmography (PPG), and electrocardiogram (ECG). These methods translate or convey the effects of myocardial activity. The second category comprises non-contact sensing setups like cardiac simulators based on imaging tools, where the manifestations of myocardial performance propagate through a medium. Contemporary non-invasive sensor-based methodologies are primarily tailored for home, remote, and continuous monitoring of myocardial performance. These techniques leverage machine learning approaches, proving encouraging outcomes. Evaluation of algorithms is centered on how clinical endpoints are selected, showing promising progress in assessing these approaches’ efficacy.

## 1 Introduction

The most recent findings from the Global Burden of Disease (GBD) study show that heart failure (HF) affects more than 64 million people globally on a spectrum of severity (Lippi and Sanchis-Gomar, 2020). Around half of all admissions for the condition are heart failure with maintained ejection fraction cases (HFpEF) (Kovács, 2015). Particularly among the elderly and those living in low-to-medium socio-demographic index (SDI) regions, HF is a developing global threat that prevalence and health loss burden continually rise. Accurate non-invasive characterization of myocardial performance is crucial to avoid sudden death.

Early recognition of heart failure is essential for underlying diseases or causes to be successfully treated and, in certain individuals, to stop further myocardial dysfunction and clinical decline. According to the Canadian cardiovascular society, suspected HF patients should firstly have a detailed clinical history as well as a physical examination to affirm or exclude myocardial dysfunction (Ezekowitz et al., 2017). Vital signs, weight, volume status, heart/lung evaluation, abdomen, and peripheral vascular assessing are crucial in evaluating the risk of heart failure. Further initial investigations are recommended (e.g., chest radiograph, ECG analysis, and lab tests). Measurements of plasma natriuretic peptides should be made if any component is abnormal. Then it is recommended to measure chamber sizes, systolic and diastolic ventricular function, valve functionality, pericardial pathology and wall thickness using two-dimensional and doppler transthoracic echocardiography. Further diagnostic investigations could be useful (e.g., cardiac catheterization, hemodynamic measurements, computed tomography (CT), cardiac magnetic resonance (CMR) imaging) when outcomes from noninvasive tests are inconclusive.

To improve the potential of heart failure, systolic and diastolic dysfunction diagnosis, classification, and characterization, the currently used non-invasive sensor-based technologies can be broadly classified into two distinct primary categories. The first group focuses on techniques that allow for a subtle investigation of cardiac dynamics through indirect patient contact (Piccini and Patrick, 2007). The ballistocardiogram (BCG), electrocardiogram (ECG), photoplethysmography (PPG), impedance cardiography (ICG), and other crucial techniques in this field (Aydemir et al., 2019; KAVAS and BOZKURT, 2022; Wang et al., 2022). These methods capture physiological signals coming from the cardiovascular system using a variety of non-invasive technologies. Notably, each of these methods acts as a channel for understanding and communicating the complex consequences caused by cardiac activity. For example, the ballistocardiogram measures the minute fluctuations in bodily motion brought on by the heartbeat, and impedance cardiography measures the changes in thoracic impedance brought on by the heartbeat. Like how the ECG detects electrical activity, photoplethysmography records changes in blood volume.

The second category includes methodologies operating under a non-contact paradigm, thus avoiding direct physical interaction with the patient. This compilation of techniques encompasses an array of advanced approaches, each contributing to a refined understanding of cardiac dynamics (Tavakoli et al., 2012; Ursani et al., 2015; Vannelli et al., 2015). Alongside magnetic resonance imaging (MRI), other pivotal cardiac imaging modalities, such as echocardiography, computed tomography, and nuclear imaging, augment this repertoire. This assortment stands as a testament to the profound innovation fueled by contemporary medical technology. Within these intricate contexts, the expressions of myocardial performance traverse intermediary mediums, facilitating insightful examinations. For example, in the realm of magnetic resonance imaging, the heart’s intricate spatial and temporal dynamics are meticulously captured and artfully reconstructed through adroit manipulation of magnetic fields and radiofrequency pulses. Similarly, echocardiography employs sound waves to visualize cardiac structures and movements. Additionally, cardiac simulators are designed to replicate the intricate anatomical and physiological attributes of the heart. These phantoms are often endowed with sensors that emulate the behavior of authentic cardiac sensors, encompassing entities like ECG and pressure sensors for hemodynamic evaluations. Through the integration of these sensors into the phantoms, researchers are empowered to rigorously validate and scrutinize the precision, sensitivity, and dependability of sensors within an environment characterized by meticulous control and replicability. Heart phantoms, designed to simulate cardiac dysfunction with the aid of imaging tools, are regarded as an innovative category of noninvasive sensing technologies, contributing significantly to the domain of heart failure modeling. By integrating sensors that mimic authentic cardiac sensors, these phantoms facilitate the evaluation and refinement of sensor-based technologies. Through the intricate interaction of imaging modalities and simulated cardiac dynamics, they afford researchers the ability to observe and analyze the complexities of heart failure without necessitating invasive interventions (Tavakoli et al., 2012; Zhu et al., 2014). This convergence of imaging techniques and sensor integration underscores the pivotal role of Heart phantoms as effective noninvasive tools for understanding and modeling heart failure scenarios, thereby advancing diagnostic precision and treatment strategies.

These simulated manifestations enable the intricate exploration of cardiac performance without needing direct interaction with a living subject by simulating real-world conditions with a controlled platform. Moreover, increasing the capacity to generate synthesized data mirroring cardiac activities under assorted conditions. This synthetic data can be harnessed to cultivate and train machine learning algorithms earmarked for the analysis of sensor data. Such endeavors notably contribute to the advancement of non-invasive methodologies pertaining to cardiac monitoring through the lens of contemporary artificial intelligence tools.

Diverse levels of models replicating the systolic and diastolic heart failure have been introduced so far. Many researchers built up heart models to analyze the hemodynamic variations in the systolic and diastolic dysfunction including ejection fraction, stroke volume, blood volume, peripheral resistance, and cardiac output. The tissue level models study muscular contractility, left ventricle twisting/torsion, and myocardial strain (Mannhardt et al., 2016; Lind et al., 2017). Whereas in the cellular level, researchers used multi-biomarker strategies, such as the endothelin, neuregulin, and TGF-β (Tian et al., 2023).

The clinical efficacy of myocardial and other cardiovascular biomarkers in the context of heart failure (HF) has been linked. However, due to their limited cardiac and HF specificity, many of these biomarkers do not meet the established criteria for prescription, as stipulated by relevant standards (Sarhene et al., 2019). Moreover, small animals were employed by many studies to examine the pathogenesis of HF and create new treatments that may halt the development of this common and deadly condition, such as using rats (Smits et al., 1992; Sakai et al., 1996) and mice (Culjat et al., 2010; Anderson, 2016). These models are highly dependent on the established heart failure and innovative molecular techniques and limited by ethical laws. As such, mathematical methodologies have supplied substantial aid in the analysis of heart failure (Bucelli et al., 2023). Notably, contemporary advancements in artificial intelligence have proven considerable potential in the realms of detection and categorization of this prevailing cardiac ailment. Concurrently, these developments have prompted prominent researchers to create mechanical simulators tailored to replicate the intricacies of cardiac chambers and vasculature. These simulators, meticulously designed to align with established imaging techniques, serve as invaluable aids in enhancing the diagnostic capabilities for heart failure. These phantoms are intended for a variety of cardiac imaging development and validation experiments, researching the variables that affect the mechanical contraction and electrical activation in the normal and pathological heart situations, and therapeutically applicable functional predictions and understanding of disease causes (Niederer et al., 2009). For the treatment of patients and for research purposes, medical imaging techniques can be extremely insightful in non-invasively quantifying the heart functionality (Gabrani-Juma et al., 2017), dynamic models for the heart were previously introduced using the SPECT (single photon emission computed tomography) algorithm for reconstruction, that estimated the dynamic nature of activity inside an object as determined by projection data obtained with a standard SPECT camera’s single slow rotation (Celler et al., 2000; Farncombe et al., 2000).

Various non-invasive sensing technologies used to characterize myocardial performance; we conducted a review of the field to outline the non-invasive sensor technologies and minimally invasive techniques employed for heart failure diagnosis, compare current practices, explain model limitations, and pinpoint knowledge needs for future study. The research inquiries pertain to the exploration of whether non-invasive sensor-based technologies exhibit adequacy in detecting heart failure, and systolic/diastolic dysfunction. Furthermore, the study seeks to elucidate the potential role of heart phantoms in enhancing the laboratory’s ability to detect heart failure.

## 2 Overview of the systolic and diastolic heart failure

The pathological state of heart failure results in less effective blood pumping. As the metabolizing tissues require it to, or only possible with elevated filling pressure. Heart failure diagnosis usually consists of the following: echocardiogram (EKG) or transthoracic echocardiogram (TTE), radionuclide ventriculography or radionuclide angiography (MUGA scan), cardiac computed tomography (CCT) scan, blood tests, such as natriuretic peptide tests, electrolyte panel and cardiac catheterization (Chatterjee and Massie, 2007; Fukuta and Little, 2008a).

In systolic heart failure ejection fraction is decreased (HFrEF) since the heart cannot contract normally. Conversely, with absence of a decreased EF, diastolic heart failure pathological situation is detected when the heart cannot fill adequately. The anatomical characteristics of the left ventricle (LV) are identical for systolic and diastolic dysfunction, increasing mass of left ventricle and LV end-diastolic pressure are two examples. Although symptoms, prognosis, and signs are very similar; the most obvious distinction between both types of heart failure is left ventricle function and geometry; systolic heart failure is defined by dilatation of left ventricle, eccentric hypertrophy of LV, and unusual systolic and diastolic functioning, However, semi-circular LV hypertrophy, a normal EF, and variable diastolic performance are the hallmarks of diastolic dysfunction (Fukuta and Little, 2008b; De Keulenaer and Brutsaert, 2011).

The paradigm of using ejection fraction (EF) to categorize HF evolved when studies in the early 2000s revealed a bimodal pattern of EF throughout HF patients (Fonarow et al., 2007). The European Society of Cardiology (ESC) of HF advocated ternary classification of heart failure: heart failure with preserved, mid-range, and reduced ejection fraction (HFpEF) (HFmEF) and (HFrEF) respectively, characterized by EF ≥ 50% (Kiranyaz et al., 2016; Sudarshan et al., 2017; Attia et al., 2019; Acharya et al., 2019; Baldoumas et al., 2019; Cheng et al., 2020; Lih et al., 2020; Thakkar and Talwekar, 2022; Kavas et al., 2023; Koh et al., 2017), %, and ≤40% respectively. From signs and symptoms of ejection fraction and heart failure, the elevated natriuretic peptides (NPs) are needed in the diagnosis of HFpEF and HFmrEF. In addition to some functional cardiac diseases, such as left atrial enlargement, LV hypertrophy and dilation dysfunction. However, characterizing the HF classification depends on the sex, age, detecting of some diseases, such as hypertension, diabetes, obesity, ischemic heart dysfunction and atrial fibrillation (Anker et al., 2018; Sarhene et al., 2019).

## 3 Non-invasive sensing technologies used for heart failure, systolic and diastolic dysfunction modeling

Non-invasive sensing technology used in modeling cardiac abnormalities can consist of distinct types of heterogeneous sensors. The various sensor configurations depend on the sort of sensing technology used (Corazza et al., 2022). These methods (Table 1) could be set up with either indirect contact with the patient (e.g., ballistocardiogram (BCG), impedance cardiography (ICG), photoplethysmography (PPG), and electrocardiogram (ECG), that can translate or transfer the impact of myocardial activity. Or set up with no-contact to the patient (e.g., heart phantoms based on imaging tools) in which the impact of myocardial performance can be propagated through a medium.

**TABLE 1:** The various sensor configurations depend on the sort of sensing technology utilized. Setup with either indirect contact with the patient (e.g., ballistocardiogram (BCG), impedance cardiography (ICG), Photoplethysmography (PPG), and electrocardiogram (ECG)). Or setup with no-contact to the patient (e.g., heart phantoms based on imaging tools). Comparing Non-Invasive Sensing Technologies.

Non-invasive methodology	Measured parameters	Applications	Advantages	Disadvantages and limitations
**Ballistocardiogram (BCG)**	- Acquiring the reaction of the pushed-out blood when the heart beats using load sensors	- Assess the clinical status of HF patients Aydemir et al. (2019)	- can be used in home monitoring	- Motion artifact signal
		- Illustrate the differences between a typical HF subject and a typical healthy subject Conn et al., 2019); Chang et al. (2020); Herkert et al. (2021)	- Designed to be wearable or with no contact with the patients	- Sensors modality mismatch
		- Early detection of HF Despins et al. (2020); Zhang et al. (2021)		
		- demonstrated the contribution between the respiratory and the cardiac system’s performance in healthy and HF populations Semiz et al. (2019)		
**Impedance Cardiography (ICG)**	- Assessing the electrical characteristics in the thorax biological tissues using two inner and two outer electrodes	- Continuous monitoring of systemic vascular resistance, stroke volume, cardiac output, and systolic time intervals Lopes et al. (2019); Avci et al. (2020); Kurpaska et al. (2022); Wang et al. (2022)	- Provide cardiovascular variables on a beat-by-beat basis	- The patient movement or postural change
				- Electrode placement
				- Blood composition
				- Skin moisture
				- Environmental radiofrequency, and even body composition
**Photoplethysmography (PPG)**	- Measuring the volumetric fluctuations in blood circulation by using a light source and a photodetector at the skin’s surface	- Evaluation of several cardiovascular-related disorders, including atherosclerosis and arterial stiffness	- Do no’t require special training or guidance	- Designed to be wearable; not ideal for long-term monitoring
		- Can help with the early detection and classification of heart failure patients Baldoumas et al. (2019); Cheng et al. (2020); Shah et al. (2020); KAVAS and BOZKURT, (2022); Thakkar and Talwekar, (2022); Kavas et al. (2023)		- Motion artifact signal
**Electrocardiogram (ECG)**	- Recording the electrical activity of the heart using electrodes fixed on the patient’s skin	- Diagnosis and evaluation of many cardiovascular disorders, including atrial fibrillation, myocardial infarction, premature contractions of the ventricles or atria, and congestive heart failure Kiranyaz et al. (2016); Acharya et al. (2017); Andreotti et al. (2017); Ghiasi et al. (2017); Sudarshan et al. (2017); Liu et al. (2018); Attia et al. (2019); Acharya et al. (2019); Al Rahhal et al. (2019); Attia et al. (2019); Lih et al. (2020); Wang and Li, (2020); Alkhodari et al. (2021)	- Do no’t need lengthy settling times, allowing for the quick acquisition of significant readings following startup	- Designed to be wearable; not ideal for long-term monitoring
				- Motion artifact signal
**Heart phantoms based on imaging tools**	- Imaging tools within heart phantoms can replicate tissue properties, blood vessel properties, mechanical strain and deformation, valve area, regurgitation, and stenosis severity	- Investigation and validation of cardiac motion approaches linked to medical image processing, reconstructing programs, and interventional guidance/tracking apps	- Realistic replication	- May oversimplify the intricate and dynamic nature of real cardiac physiology
		- Pulsatile flow simulation to validate the hemodynamic force data	- Noninvasive exploration	- Provide static representations of cardiac conditions
		- Create a dynamic physical heart phantom with an accurate coronary plaque to test the accuracy of stenosis assessment	- Repeatable and customizable experiments	- Cost and complexity: Developing, maintaining, and operating heart phantoms with advanced imaging capabilities can be resource-intensive, making them less accessible for smaller research institutions
			- Sensors integration, allowing for various parameters measurement	
			- Enable the validation of imaging modalities’ accuracy and reliability in capturing cardiac structures and dynamics	

Ballistocardiogram (BCG): is a technique of acquiring the reaction of the pushed-out blood when the heart beats. Impedance cardiography (ICG): assessing the electrical characteristics in the thorax biological tissues. Photoplethysmography (PPG): a wearable optical tool that is used non-invasively in blood circulation to measure volumetric fluctuations. Electrocardiogram (ECG): records the electrical activity of the heart over a period of time. Heart phantoms based on imaging tools: physical or virtual models designed to replicate the anatomical and functional features of the human heart, particularly in the context of cardiovascular imaging and diagnostic techniques.

### 3.1 Ballistocardiogram (BCG)

Noninvasive and unobtrusive vital sign sensing is made possible by technological developments in hardware and software. BCG is a technique of acquiring the reaction of the pushed-out blood when the heart beats, these vibration signals are indirectly detected using load sensors fastened to a chair or bed. The primary challenge is how to improve the use of BCG technology, with a focus on varies devices used for measurement, and signal processing approaches. Diastolic and systolic blood pressure monitoring based on BCG was adapted, showing good opportunities for cuff-less pressure measurements (Kim et al., 2018). BCG signals of good quality were detected at home setting after discharge and analyzed to assess the clinical status of HF patients (Aydemir et al., 2019). This work reported 0.78 of area under the curve (AUC) score for the classification task. Another in home HF monitoring technology were introduced by researchers in (Conn et al., 2019), they used toilet seat–based BCG model to illustrate the differences in the averaged waveforms collected from heart failure and healthy subjects, while they were at rest and post-stressed. BCG is used with the ECG to estimate the stroke volume and measure blood pressure by finding the beginning of the transit pulse time.

Early HF changes were shown in a female older adult using BCG sensors embedded under a bed mattress (Despins et al., 2020), the analyzed waveforms indicated cardiac output reduction as the diastolic performance worsened. For the resting state, BCG waveforms of healthy and HF patients were compared (Chang et al., 2020). In this study, the waveform fluctuation metric at rest (WFMR) were calculated for both cohorts, acquired from an instrumented chair with four load cells. By using algorithms, there may be more errors, since the results revealed that the heart failure set had more fluctuations compared to the healthy subjects with at least 82.2% separation. Furthermore, tele-monitoring changes in hemodynamic load of fifteen patients with HFrEF were investigated in this study (Herkert et al., 2021). They used a device made up of BCG sensor implemented in lower back configuration, that acquires the body micro movements, as blood inflow, and chest sensor, which collects local thoracic fluctuations resulted from cardiac contraction and blood ejection through great vessels [seismocardiography (SCG)]. Those collected kinocardiographs (KCG) are converted to cardiac maximum power (Pmax) integral, cardiac kinetic energy (iK), and change in cardiac kinetic energy (ΔiK), making it encouraging for future remote monitoring of heart failure. Significant increases in the BCG iK and BCG Pmax over the cardiac cycle (CC) were found, while SCG iK and Pmax were revealed in a non-significant elevation. Another work confirmed that KCG have potential to detect HF patients (De Keyzer et al., 2023), As SCG ΔiK diastolic, BCG ΔiK diastolic, and BCG iK systolic found to be significantly different between HF patients (EF < 50) and normal patients (EF ≥ 50).

Feng et al. used a non-contact piezoelectric sensing device and the echocardiography to detect HF patients with LVEF ≤49 (Feng et al., 2023). Through the extracted linear and nonlinear BCG features, cardiopulmonary, and respiratory features, they demonstrated the contribution between the respiratory and the cardiac systems performance in healthy and HF populations. The detection of HF patients was finally done through XGBoost classifier with an accuracy of 94.97%. In another work (Semiz et al., 2019), they demonstrated the BCG signals’ potential for monitoring hemodynamic reactions to dose adjustments of diuretics in HFrEF patients. In this study, 12 BCG features were recorded using a modified-weighing scale in addition to 6 extracted features from ECG signals. Elevated left atrial pressure (LAP) is considered as a HF indicator in this work (Zhang et al., 2021), they collected thoracic vibration signals through the optical BCG device that is fixed on each patient’s back, then verified the left atrial pressure index with a 0.81 overall agreement rate as a derived parameter for heart failure detection.

### 3.2 Impedance cardiography (ICG)

It is recognized as a diagnostic method for assessing the electrical characteristics in the thorax biological tissues. In this technique, two outer electrodes are used to provide a small, alternating, constant current that has an amplitude between 1 and 5 mA and a frequency between 20 and 100 kHz. Two inner electrodes are then used to measure the electrical voltage difference (Woltjer et al., 1997). ICG limitations include the patient movement or postural change, electrode placement, blood composition, skin moisture, environmental radiofrequency, and even body composition. Nevertheless, it is considered a reliable technology used to noninvasively estimate the cardiovascular hemodynamic pattern. ICG has the potential to provide a beat-by-beat variables in the cardiovascular system. Some of its potential clinical applications include continuous monitoring of systemic vascular resistance, cardiac output, stroke volume, and time intervals of the systolic activity. Authors in (Facchini et al., 2016) suggested a multi-parametric noninvasive hemodynamic approach to identify the pulmonary congestion in chronic HF patients. They observed significant relation between B-line number, levels of brain natriuretic peptides (BNP) and ICG. In this work, the inverse of impedance (thoracic conductance) was measured using four sensor pairs made of silver-silver chloride: two in the mid-axillary line on each side of the chest at the level of the xyphoid, and two under each ear at the neck base.

In another study (Lopes et al., 2019), researchers used impedance cardiography to compare the hemodynamic parameters of hypertensive patients in a population sample with and without heart failure. Four pairs of ICG electrodes were fixed on the lower chest and on the subject’s neck. After analyzing the recorded indices, it is found that the mean values of the inotropic state index (ISI), ejection phase contractility index (EPCI) and left stroke work index (LSWI) are reduced in heart failure patients. Furthermore, ICG technology were used to measure hemodynamic parameters to investigate the phase I cardiac rehabilitation (CR) effect on subjects with acute heart failure (AHF) and coronary heart disease (CHD) (Wang et al., 2022). Results in this work showed ameliorating in preload, systolic and diastolic functionality improvements, elevating cardiac output, and afterload relieving.

However, researchers in (Kurpaska et al., 2022) targeted the coronary artery disease (CAD) patients to study the correlation between the ICG hemodynamics and the cardiopulmonary exercise testing (CPET) parameters. Where a clinically meaningful increase in cardiac output (CO) following exercise was caused, to varying degrees, by changes in stroke volume (SV) and heart rate (HR).

Thoracic fluid content (TFC) is obtained using the ICG device from heart failure patients with dyspnea and are suspected of pulmonary oedema (PO) (Avci et al., 2020). They captured TFC by attaching electrodes in the lower and longitudinal thorax. Voltage is produced when the body is exposed to a 400 µA steady current at 40 kHz between the electrodes. Thoracic voltage variations are detected using inside electrodes. The impedance change is computed using these voltage changes. This work revealed that TFC could be used significantly in the diagnosis of PO, with sensitivity/specificity of 64.3/31.4%.

### 3.3 Photoplethysmography (PPG)

PPG is a wearable optical tool that is used non-invasively in blood circulation to measure volumetric fluctuations. This is achieved by a light source and a photodetector at the surface of the skin. Important health-related information is contained in PPG wave (e.g., vascular dynamics, oxygenation, heart rate, and rhythm). To evaluate several cardiovascular-related disorders (e.g., arterial stiffness and atherosclerosis), can thus, be achieved by the examination of this waveform for doctors and researchers. Also, analyzing the PPG second derivative signal can be helpful in early diagnosis and detection of different cardiovascular diseases that could potentially manifest in life (Castaneda et al., 2018). While resting in a chair, reflective PPG and 3-axis accelerometry signals were captured from patients for 5 min to evaluate the pressure-related mechanisms and baroreflex of the heart (Shah et al., 2020). PPG signals were then used to calculate the standard metrics of heart rate variability (HRV). Furthermore, from the calculated data, they classified individuals with HF using linear support vector machines (SVMs) at an accuracy of 74%.

In another work, by using only heart rate variability (HRV) collected from photoplethysmography, machine learning model was enhanced to detect HFrEF and HFpEF (KAVAS and BOZKURT, 2022). Volunteers provided PPG data for 10s, which were then preprocessed using digital filters and analyzed to evaluate thirty-seven HRV characteristics. Classification was done with 3 crucial features retrieved from applying 10-fold cross validation of %98.33 accuracy. In addition, another group of researchers used PPG to estimate 58 significant HRV features based on Mann Whitney-U Test (Kavas et al., 2023). Then using these features, triple classification model (healthy, HFpEF, and HFrEF) was evaluated using 10-fold cross validation at accuracy of %87.78.

Blood pressure estimation (BPE) is another significant evaluation from the recorded PPG signal (Thakkar and Talwekar, 2022). Researchers proposed a deep network model based on the BPE to classify patients into four groups: stroke, heart failure, aneurysm, and heart attack. They used an improved differential algorithm (IDA) to calculate three categories of BPE from PPG signal as prehypertension, hypertension, and normotension. Then significant features were fed to a deep network classifier that performed maximum accuracy of 97.80%.

In another work, using data augmentation 10s PPG signals were converted into a 128 × 1,024×3 time-frequency chromatograph (Cheng et al., 2020), then they used it to train and validate a framework of hybrid combination of convolutional neural network (CNN) and long short-term memory (LSTM) for classifying patients to be with or without atrial fibrillation (AF) at accuracy of 98.21%. Baldoumas et al. (Baldoumas et al., 2019), proposed a portable model of PPG device to detect individuals with cognitive heart failure from the healthy, they used natural time analysis (NTA) method to analyze and classify the series of sequential events collected from the PPG device.

### 3.4 ECG data and AI-derived HF models

One of the most popular non-invasive diagnostic techniques for monitoring the heart’s physiological processes throughout time is the electrocardiogram (ECG). Many cardiovascular disorders, including atrial fibrillation, myocardial infarction, premature contractions of the ventricles or atria, and congestive heart failure, can be diagnosed with the help of ECG data. Wearable technology in the healthcare industry, including the Apple Watch and portable ECG monitors like the Holter monitor, have both developed quickly in recent years. As a result, human cardiologists are struggling to keep up with the growing volume of ECG data that needs to be analyzed. Consequently, automatic and accurate ECG data analysis has become a popular study area, especially using AI tools. The diagnostic golden rules have been used for automatic ECG analysis. This process consists of two steps, that involve human specialists to create meaningful features from raw ECG data, known as “expert features,” and then to come at inferences, using prediction model or other machine learning approaches. These features could be categorized into coefficients of variation and density histograms, statistical features (e.g., heart rate variability), frequency-domain features, time-domain features and sample entropy.

However, due to the limitations imposed by human expert knowledge and data quality, they are still insufficient. Alternatively, deep learning models use their robust data learn to automatically and implicitly conduct feature extraction, and model heart failure. Most recent AI heart failure models based on ECG data is summarized in Table 2. Authors in (Sudarshan et al., 2017) proposed decision tree (DT) and k-nearest neighbor (KNN) classifiers for the identification of ECG signals showing congestive heart failure (CHF) from healthy. The one lead ECG signal of 2 seconds length was converted with the dual tree complex wavelets transform (DTCWT), then features were retrieved statistically and ranked using entropy, Bhattacharyya, receiver-operating characteristics (ROC), *t*-test, minimum redundancy maximum relevance (mRMR), random forest (RF), and Wilcoxon methods. And classification accuracy of 99.86% was reported in this study. Whereas in (Acharya et al., 2019), CHF diagnosis model-based on 2 seconds one lead ECG data was proposed. With no features extracted, the deep convolutional neural network (CNN) of 11 layers achieved detection accuracy of 98.97%. In another study (Lih et al., 2020), 2-s one lead ECG (2000 sample) window length data were used to classify ECG signals into myocardial infarction (MI), coronary artery disease (CAD) and CHF, with overall accuracy of 98.51% using the CNN-LSTM (16 Layers) tool.

**TABLE 2:** Deep learning models use their robust data learn to automatically and implicitly conduct feature extraction, and model heart failure. Most recent AI heart failure models based on ECG data is summarized. AI Heart Failure models based on ECG data.

Study	Signal processing & featuring technique	AI classification method	Sample Size	Study Outcome	Classification performance
(Sudarshan et al., 2017)	1. Dual tree complex wavelets transform (DTCWT) of 2 s one lead ECG.	k-nearest neighbor (KNN) and decision tree (DT) classifiers	88 Patients	Identification of ECG signals exhibiting CHF from normal	Accuracy 99.86%
	2. Using the Bhattacharyya, entropy, minimum redundancy maximum relevance (mRMR), receiver-operating characteristics (ROC), Wilcoxon, *t*-test, and random forest (RF) methods, statistical features are retrieved and ranked				Sensitivity 99.78%
					Specificity 99.94%
(Acharya et al., 2019)	1. No denoising required	11-layer deep convolutional neural network (CNN)	88 Patients	Diagnose CHF	Accuracy 98.97%
	2. No Features extracted				Sensitivity 98.87%
	3. 2s one lead ECG regularized with Z score normalization				Specificity 99.01%
(Lih et al., 2020)	1. 2-s one lead ECG (2000 sample) window length	CNN-LSTM (16 Layer)	Normal: 92	Classification of ECG signals into CAD (Coronary artery disease), MI(Myocardial infarction), and CHF(Congestive heart failure)	Accuracy 98.51%
			CAD: 7		Sensitivity 99.30%
			MI: 148		Specificity 97.89%
			CHF: 15		
Attia et al. (2019)	10-s 12-lead ECG	CNN trained using the Keras framework with a backend and (Python) Tensorflow (Google)	3,874	Identification of EF less than or equal to 35%	Accuracy 86.5%
					Sensitivity 82.5%
					Specificity 86.8%
Kiranyaz et al. (2016)	1. 30-min duration of two-channel ECG signals	CNN layers of 3 × 3 kernels (Kx = Ky = 3)	44 ECG records	ECG beats classification	Accuracy 98.9%
	2. Band-pass filter at 0.1–100 Hz and then binaries at 360 Hz				Sensitivity 95.9%
	3. R-peak detection then FFT representation (phase and magnitude) of each beat				Specificity 99.4%
Liu et al. (2018)	1. The ECG signal is split to isolate the heartbeats	Multilead-CNN	549 records from290 individuals	Myocardial Infarction (MI) detection	Accuracy 96%
	2. Fuzzy Information Granulation (FIG) is performed for each heartbeat to determine 3 parameters a, c, and m				Sensitivity 95.4%
					Specificity 97.37%
Acharya et al. (2019)	1. Using Daubechies wavelet 6 filters, denoise and remove the baseline from each and every ECG signal	CNN	47 subjects	Detect five classes of ECG heartbeats	Accuracy 93.47%
	2. Signals that have been divided into beats, beats that are centered around R-peaks, and R-peak detection using Pan-Tompkins		From ECG signal Lead II.		Sensitivity 96.01%
	3. To address the issue of amplitude scaling and get rid of the offset effect, normalized using Z-score normalization				Specificity 91.64%
Andreotti et al. (2017)	Feature-based approach	Residual Network architecture	8,528 short single lead ECG segments	Classify ECG into 4 classes (normal, AF, other rhythms or noise)	Final score for two approaches: F1 = 79%
	1. Cut-off frequencies for 10th order bandpass Butterworth filters are 1Hz–100Hz and 5Hz–45 Hz (narrow band) (wide band)				
	2. QRS detectors: pqrs, PanTompkins (jqrs), maxima search, and matched filtering. classical				
	3. Time domain, frequency domain, and non-linear HRV, clustering of beats on Poincare plots				
Wang and Li, (2020)	1. Bandpass filter denoised the original signal (3Hz–45 Hz)	CNN and LSTM	8,258 one channel individual recordings	Classify atrial fibrillation and normal sinus signals	F1 score of 0.82
	2. Dual-slope QRS detection algorithm for detecting R peaks and calculating R-R interval				
	3. Divide into segments containing at least 4 R-R intervals				
Al Rahhal et al. (2019)	2D image using a generative neural network	Dense Convolutional Networks (DCN)	48 of two-channel ECG recordings	Classification of electrocardiogram beats	Sensitivity 73%–99% depending on the used method
Attia et al. (2019)	Echocardiogram and ECG of 12-lead data	CNN using the Tensorflow (Google) backend Python and Keras framework	44,959 patients	Identify patients with ventricular dysfunction EF ≤ 35%	Accuracy 86.3% Sensitivity 85.7%
					Specificity 85.7%
Ghiasi et al. (2017)	1. Baseline wander removal using Butterworth low and high pass filter	CNN	8528 ECG recordings	Atrial fibrillation detection	Featuring Approach scores 78%
	2. T, QRS complex and P are detected using the classical pan Tompkins				CNN Approach scores 71%
	3. Z-score normalization				
	4. [Morphological features (fractal dimension, correlation coefficient, and R peaks variance) and time/frequency domain features] used for Featuring Approach				
Alkhodari et al. (2021)	1. HRV pre-processing and denoised for every hour through out 24-h cardiac cycle	Jenks natural break optimization algorithm	24-h Holter ECG recordings of 92 patients	Classify HF patients depending on the EF levels (HFpEF, HFrEF and HFmEF)	Accuracy more than 70%
	2. HRV feature extraction and HRVEF index estimating				

Moreover, to identify patients of EF less than or equal to 35%, researchers in (Attia et al., 2019) trained the CNN framework using the 10-s 12-leads ECG data. They reported overall accuracy of 86.5% in this model. For ECG beats classification, 30-min duration of two-channel ECG signals were used in this work (Kiranyaz et al., 2016). After band-pass filtration at 0.1–100 Hz and then signal binarization at 360 Hz, R-peaks were detected, then represented using FFT (phase and magnitude) for each beat. The used CNN model reported an accuracy of 98.9%. In another work (Liu et al., 2018), ECG signal is split to isolate the heartbeats before performing fuzzy information granulation (FIG) for each heartbeat to figure out 3 parameters a, c, and m. Those heartbeat parameters used to detect the myocardial infarction, their multimodal-CNN model reported detection accuracy of 96%. Moreover, researchers in (Acharya et al., 2017) detected five classes of ECG heartbeats based on ECG signal Lead II. After Daubechies filtration, de-noise and remove the baseline from each ECG, signals have been divided into beats that are centered around R-peaks, then R-peak detection performed using Pan-Tompkins. This CNN detection model reported an accuracy of 93.47%. ECG feature-based approach was introduced by (Andreotti et al., 2017) for ECG classification into 4 classes (AF, normal, other rhythms or noise). Firstly, they preprocessed the short single lead ECG segments using Butterworth bandpass filters of 10th order, with cut-off frequencies of 5Hz–45 Hz (narrow band) and 1Hz–100 Hz (wide band). Then after QRS detection, classical time domain, non-linear HRV, and frequency domain features were detected and further fed to residual network architecture. This classification approach reported a final score F1 of 79%.

In another study (Wang and Li, 2020), one channel ECG individual recordings were used to classify atrial fibrillation and normal sinus signals. This CNN and LSTM-based model achieved an F1 score of 0.82. The original signal was filtered with bandpass filter of 3Hz–45 Hz bandwidth. Then they used dual-slope QRS detection algorithm for detecting R peaks and calculating R-R interval, where each segment was divided to contain at least 4 R-R intervals. Another study (Al Rahhal et al., 2019) proposed an approach to classify ECG beats based on two-channel ECG recordings. 2D image using a generative neural network was generated and fed to the dense convolutional networks (DCN), that achieved a classification model with maximum sensitivity of 99%. To find patients with ventricular dysfunction EF ≤ 35%, researchers in (Attia et al., 2019) used both echocardiogram and ECG of 12-lead data. This CNN-based model reported an accuracy of 86.3% using the tensorflow (Google) backend Python and Keras framework.

Furthermore, 8528 ECG recordings were used by researchers in (Ghiasi et al., 2017) for atrial fibrillation detection. For the featuring approach they detected morphological features (e.g., fractal dimension, correlation coefficient, and R peaks variance) and time/frequency domain features. The reported scores for the featuring approach and the CNN model were 78% and 71%, respectively. An approach based on heart rate variability was proposed by researchers in (Alkhodari et al., 2021), they aimed to categorize heart failure patients into three groups according to LVEF levels (HFpEF, HFrEF, and HFmEF) during the 24-h circadian cycle. Adaptive filtering and signal dependent rank order mean (SD-ROM) were firstly implemented to 24-h HRV data, then Jenks natural break optimization algorithm was performed to generate heart rate variability ejection fraction index (HRVEF) groups. This approach accurately reported more than 70% goodness of variance fit (GVF) during the time ranges of (01:00-08:00) and (17:00-23:00).

### 3.5 Heart phantoms simulating cardiac dysfunction based on imaging tools

One of the main categories of heart phantoms is designed to suit imaging applications specially for studying the cardiac dysfunction. Imaging simulators play significant role in assessing imaging systems. By nature, phantoms deliver reliable and repeatable results, allowing efficient methods and systems comparisons, unlike depending on data collected from live subjects for test purposes (Sakai et al., 1996; Culjat et al., 2010). Besides, it limits ethical issues concerning human and animal testing (Sakai et al., 1996).

Recently, researchers in (Vannelli et al., 2015) designed a phantom that replicates the motion of the aortic and mitral valves *in vivo*, enabling the acquisition of accurate ultrasound pictures of these parts. Additionally, it possesses a physiologically plausible 50% left ventricular ejection percent. Heart phantoms were used widely to mimic the cardiac mechanics and structure. A biventricular polyvinyl alcohol (PVA) based heart phantom was created that resembles the form of the heart including the characteristics of MRI and ultrasound imaging. Further phantom with pathologic characteristics that mimicked an aneurysm and scarred areas was used (Tavakoli et al., 2012). PVA polymers that are more rigid were used to replicate the scarred tissue. Realistic asymmetric LV motion is made possible by the two-chamber cardiac structure. Unhealthy heart rhythms were dynamically simulated (Ursani et al., 2015), the dynamic heart phantom can be actuated by a built in simulator or by using real time ECG signal for the patients, which can then be scanned in the CT to determine the appropriate gating strategy to the patient’s present heartbeat. A patient’s data with cardiac arrhythmia were used to build a physical heart phantom (Sandoval et al., 2018), where transesophageal therapy for atrial fibrillation is guided by an adjusted registration strategy.

As described in Table 3, a brief comparison of some of the phantoms and the used imaging technique is shown. An *in vitro* protocol was carried out using right ventricle (RV) shaped phantoms composed of various materials that could be imaged using cardiac magnetic resonance (CMR), cardiac computed tomography (CT) and RT3DE (Sugeng et al., 2010). They manually controlled this phantom to evaluate the RV volume. In another work (Tavakoli et al., 2012), researchers proposed a pathologic heart phantom to mimic an infarction and an aneurysm. Three inclusions with varying forms and degrees of flexibility were used to represent segmental dyskinesia. They manually controlled the PVA cryogen’s freeze-thaw cycles and used MRI and ultrasound for imaging. Applying the B-spline deformable image registration method (BSDIR) a series of long-axis labeled MR images were used to estimate the MRI strains (Zhu et al., 2014). This approach was tested against Shelley’s dynamic to investigate and validate cardiac motion linked to medical image processing, reconstructing programs, and interventional guidance/tracking apps.

**TABLE 3:** Heart phantoms are designed to suit imaging applications specially for studying the cardiac dysfunction. Imaging simulators play significant role in assessing imaging systems. This table summarizes the dynamic hearts modeling myocardial dysfunction. List of previously developed heart phantoms based on imaging tools.

References	Heart phantom control	Ventricular pressure and heart rate variability	Imaging tool	Objective
Vannelli et al. (2015)	Arduino controlled	Pressure sensors fixed to record aortic, ventricular and atrial pressures at fixed heart rate set to 60bpm	Ultrasound Views	Create tools for picture guiding in valve repair and replacement. Having a 50% left ventricular ejection fraction
Sugeng et al. (2010)	Manual control	An *in vitro* protocol was carried out using RV-shaped phantoms composed of various materials that could be imaged using various imaging techniques. while pressure and heart rate were not investigated	Cardiac computed tomography (CT) and cardiac magnetic resonance (CMR), RT3DE.	On CMR, CCT, and RT3DE pictures, volumetric quantification of RV volume was tested
Tavakoli et al. (2012)	Manual control	Internally produced biventricular multimodal cardiac phantom that can mimic both healthy and unhealthy conditions, here the pressure and heart rate were not investigated	MRI and ultrasound imaging	Building a pathologic heart phantom to mimic an infarction and an aneurysm. Three inclusions with varying forms and degrees of flexibility are used to represent segmental dyskinesia. Depending on PVA cryogen’s freeze-thaw cycles
Zhu et al. (2014)	Modeling the heart computationally and testing it against Shelley’s dynamic	The fully nonlinear equilibrium equations are resolved using a mixed displacement/pressure formulation. It was not the hydrostatic pressure that was applied inside the LV and RV that was employed to govern the motion of the heart phantom. In tests, the heart phantom was made to beat at a rate of 60 beats per minute	A series of long-axis labeled MR images were used to estimate the MRI strains using the B-spline deformable image registration method (BSDIR)	The heart phantom was created with the specific intention of being used as a tool for the investigation and validation of cardiac motion approaches linked to medical image processing, reconstructing programs, and interventional guidance/tracking apps
Töger et al. (2018)	Peak speeds employed in the five distinct pump programs ranged from 21 to 36 cm/s	For visualization reasons, a pressure field calculation was made. Using a multigrid solver that had already been published, the pressure Poisson equation (PPE) was resolved. The study’s parameters called for healthy subjects with normal heart rates	A References flow field in the flow’s central symmetry plane was measured using a particle image velocimetry (PIV) setup made up of a Continuum MiniLite 532 nm Nd:YAG laser, a FlowMaster 3S camera (LaVision, Bicester, United Kingdom), and related controller gear	Creating software for the analysis of 4D flow MRI data for hemodynamic forces. Additionally, a pulsatile flow simulation is employed to validate the hemodynamic force data
Richards et al. (2018)	The conduit was filled with an iodinated contrast substance to imitate a typical contrast-enhanced CCTA protocol. Shelley Medical Imaging DHP-01 was used as the foundation for the heart motion implementation	Four different heart rates were fitted with the generated ECG signal, and the signals were included into the controller software	A clinical cardiac protocol was used to obtain images with a dual-source CT system (Siemens Healthineer, Somatom Definition Flash, Forchheim, Germany)	Create a dynamic physical heart phantom with an accurate coronary plaque to test the accuracy of stenosis assessment at clinically appropriate heart rates
Ursani et al. (2015)	Anthropomorphic heart phantom (DHAP) based on piston fluid pump, which can mimic the patient’s heart rhythm while patient with irregular and higher HR is waiting in the holding area	The Hazen Williams Equation is used to determine the pressure difference P) across the delivery pipe connecting the heart pump and heart module. ECG sync output interface, 50 to 125 BPM Programmable cardiac motion software components	To determine the optimal gating strategy suitable for the patient’s present cardiac rhythm AND X-ray, the patient was scanned in the CT.	The creation of a dynamic anthropomorphic heart phantom (DHAP) that can accurately replicate any patient’s heart rhythm while they wait in the holding area
Zhou et al. (2018)	Includes heart chambers, external circulation, bionic valves, a computerized control system, and a motor mechanism	When the heart rate is between 40 and 90 beats per minute, the end-diastolic and end-systolic pressures are chosen to be 2.67 kPa and 13.3 kPa, respectively. Additionally, backflows of 7.6% and 1% are found for the mitral and tricuspid valves that under heartbeat of 60 bpm	Only the phantom was CT scanned, and all of its parts can be clearly distinguished in the resultant slice images	The suggested biomimetic cardiac simulator was driven by artificial muscle
Sandoval et al. (2018)	A commercial heart model (PVAH-01 Medical Imaging Technologies, Shelley Automation Inc. London, Canada) limited to the ventricles	This phantom’s size is comparable to a woman’s heart, with the left ventricle being 76 mL and the right ventricle measuring 66 mL. They altered this phantom to incorporate a tube-like structure that represents the esophagus	The physical phantom CT images was processed, A smoothed centerline was fitted from a set of 180 extracted points of segmented esophagus	To enhance the direction of transesophageal high intensity focused ultrasound (HIFU) atrial fibrillation therapy, create a new image-processing technique. a brand-new 2D-US to 3D-CT registration technique that was tailored for the control of arrhythmia transesophageal HIFU treatment
Chen et al. (2021)	A Dynamic Cardiac Phantom (Data Spectrum Corporation, Durham, North Carolina, United States ) was programmed to pump at EF = 30 or EF = 60.	For SPECT and CT, the heart rates were respectively 40, 60, 80, and 100 bpm	Single photon emission computed tomography (SPECT) and CT imaging	This study compares the CT and SPECT calculations of ejection fraction (EF) at high heart rate. CT and SPECT were compared using a dynamic cardiac phantom with customizable end-systolic volume (ESV), end-diastolic volume (EDV), and heart rate
Urbina et al. (2016)	An MRI-compatible pump, aortic model, control unit, shutdown valves, and compliance chamber, nonreturnables make up the phantom	Systole and diastole pressures were measured at 5 different positions in the aortic model. Normal phantom and healthy volunteers’ hemodynamic values were as follows: heart rate: 68/61 bpm, cardiac output: 3.5/4.5 L/min	MRI imaging for flow and velocity waveforms measuring	To create and evaluate an aortic phantom that is compatible with magnetic resonance imaging (MRI), modeling both normal and aortic coarctation (AoCo) circumstances and to compare its hemodynamics with healthy volunteers and AoCo patients. And improve the systole and diastole pressure gradients compared with previous studies

Furthermore, authors in (Töger et al., 2018) created software for the analysis of 4D flow MRI data for hemodynamics. Additionally, a pulsatile flow simulation was employed to validate the hemodynamic force data. Using a particle image velocimetry (PIV) the reference flow field in the flow’s central symmetry plane was measured, with setup made up of a FlowMaster 3S camera (LaVision, Bicester, United Kingdom), Continuum MiniLite 532 nm Nd:YAG laser and related controller gear. In another work (Richards et al., 2018), a dynamic physical heart phantom was created with an accurate coronary plaque to test the accuracy of stenosis assessment at clinically appropriate heart rates. In this work the conduit was filled with an iodinated contrast substance to imitate a typical contrast enhanced CCTA protocol. Shelley Medical Imaging DHP-01 was used as the foundation for the heart dynamic implementation, and four different heart rates were fitted with the generated ECG signal before the signals were included into the controller software. Then a clinical cardiac protocol was used to obtain images by a dual-source CT system (Somatom Definition Flash, Siemens Healthineer, Germany and Forchheim).

Authors in (Ursani et al., 2015) based on piston fluid pump, created a dynamic anthropomorphic heart phantom (DHAP), that accurately replicates any heart rhythm for patients waiting in the holding area. The Hazen Williams equation was used to determine the pressure difference P) across the delivery pipe connecting the heart module and heart pump. To determine the optimal gating strategy suitable for the patient’s present cardiac rhythm and X-ray, the patient was scanned in the CT and ECG sync output interface, 50 to 125 BPM programmable cardiac motion software components were used. In another work (Zhou et al., 2018), researchers suggested a biomimetic cardiac simulator driven by artificial muscle. This system included heart chambers, external circulation, bionic valves, a computerized control system, and a motor mechanism. They scanned the process using CT technique, and when the heart rate was between 40 and 90 bpm, the end-systolic and diastolic pressures were chosen to be 2.67 kPa and 13.3 kPa, respectively. Additionally, backflows of 7.6% and 1% were found for the mitral and tricuspid valves that under heartbeat of 60 bpm.

To enhance the therapy of atrial fibrillation based on the direction of transesophageal high intensity focused ultrasound (HIFU), a new 3D-CT to 2D-US registration technique was created (Sandoval et al., 2018). This phantom’s size was comparable to a woman’s heart, with the right ventricle measuring 66 mL, and the left ventricle being 76 mL. They altered this phantom to incorporate a tube-like structure that represents the esophagus. The physical phantom CT images were processed; and they fitted smoothed centerline from a set of 180 extracted points of segmented esophagus. Another study (Chen et al., 2021) compared the CT and single photon emission computed tomography (SPECT) calculations of ejection fraction at high heart rate. CT and SPECT were compared using a dynamic cardiac phantom with customizable end-diastolic volume (EDV), end-systolic volume (ESV), and heart rates of 40, 60, 80, and 100 bpm, respectively. In this work, the dynamic cardiac phantom (United States , Durham, Data Spectrum Corporation, North Carolina) was programmed to pump at EF = 30 or EF = 60.

Furthermore, researchers in (Urbina et al., 2016) aimed to create and evaluate an aortic phantom that is compatible with magnetic resonance imaging (MRI), modeling both aortic coarctation (AoCo) and normal circumstances. To compare its hemodynamics with AoCo patients and healthy volunteers, they made up the system using an MRI-compatible pump, aortic model, control unit, shutdown valves, compliance chamber, and non-returnables. In this work the systole and diastole pressures were measured at 5 distinct positions in the aortic model. Normal phantom and healthy volunteers’ hemodynamic values were as follows: cardiac output: 3.5/4.5 L/min, heart rate: 68/61 bpm, and MRI imaging was used for flow and velocity waveforms measuring.

## 4 Overall perspective

### 4.1 Implications

1. Extracted features and home monitoring: Linear and nonlinear features derived from BCG signals showed significant distinctions between normal and heart failure patients. Home monitoring technologies have embraced diverse configurations of embedded BCG sensors, indicating promising prospects for future tele monitoring of heart failure patients.
2. ICG potential: Impedance signals appear as a potential source of heart rate information through beat-by-beat analysis. Continuous monitoring of systemic vascular resistance, stroke volume, cardiac output, and systolic time intervals stands as exemplary clinical applications of this technique.
3. Significance of PPG wave: The PPG waveform encapsulates a wealth of health-related information, encompassing vascular dynamics, oxygenation levels, heart rate, and rhythm. comprehensive analysis of this waveform holds potential in diagnosing various cardiovascular disorders, including arterial stiffness and atherosclerosis
4. ECG as a diagnostic instrument: ECG reigns as a preeminent non-invasive diagnostic tool for real-time monitoring of cardiac physiological processes. Its application extends to the diagnosis of diverse cardiovascular ailments, encompassing atrial fibrillation, myocardial infarction, ventricular and premature atrial contractions, and congestive heart failure.
5. Integration of Deep Learning: Modern technological advancements harness deep learning models to autonomously and implicitly extract features from ECG data. These models further simulate heart failure based on ECG data, augmenting diagnostic capabilities.
6. Role of heart simulators: Heart simulators, adept at emulating the mechanical dynamics of the heart, yield dependable and replicable outcomes. They facilitate meticulous process and system comparisons, thereby underpinning reliable advancements in cardiac research and diagnostics.

### 4.2 Limitations

While exploring various methodologies and technologies for cardiovascular assessment, it is important to acknowledge certain limitations that appear during their application. This section highlights several constraints and challenges encountered in the implementation of these techniques.

1. Variability in BCG design: The design of the fixed template for BCG exhibits significant variability contingent on the dynamic measurement conditions. Consequently, it does not conform to a universal standard. The alterations in patient movement, postural adjustments, electrode placement, blood composition, skin moisture levels, ambient radiofrequency interferences, and even variations in body composition collectively contribute to the constraints met in ICG.
2. Challenges in Real-Time PPG: Acquiring a real-time PPG signal while mitigating considerable artifacts presents a notable challenge. The comprehensive assessment of the influence of diverse noise sources on the real-time PPG signal and the subsequent development of methodologies for their effective elimination from the acquired signal are components integral to forthcoming research endeavors.
3. ECG measurements can be influenced by factors such as electrode placement variability, patient movement artifacts, and interference from external electromagnetic sources. Additionally, while modern technology leverages deep learning models to enhance ECG data analysis, the interpretability of these models remains a challenge.
4. Heart simulators for imaging tools: The primary design orientation of heart simulators is geared towards accommodating imaging assessment tools. These simulators are characterized by their applicability to high-cost prototyping and maintenance. As indicated in the reviewed literature, the predominant focus of heart simulators lies in serving imaging methodologies, underscoring their specialized utility in this context.

### 4.3 Recommendations

Based on the analysis and findings presented in the study, to support the creation and adoption of non-invasive sensor-based technologies, foster collaboration between researchers, engineers, physicians, and industry stakeholders. Working together can assist in solving technological issues, encourage data exchange, and hastening the conversion of research into clinical practice. However, to evaluate the precision, dependability, and generalizability of sensor-based models for heart failure, systolic and diastolic dysfunction, comprehensive validation studies involving various patient populations should be carried out. To guarantee the clinical relevance and efficacy of these technologies across various patient profiles and healthcare contexts, robust validation is essential.

Also, concentrate on the creation of wearable sensors and remote monitoring systems that can track important physiological characteristics over a long time. Long-term monitoring can offer insightful information about the course of the disease, the effectiveness of the treatment, and the early identification of exacerbations, allowing for prompt interventions and individualized care. Devote close attention to how user-friendly and satisfying non-invasive sensor-based solutions are; create mobile applications with user-friendly interfaces and clear instructions so that patients and healthcare professionals can quickly explore and comprehend the gathered data. Patients can be empowered to participate in their own care and make educated decisions with clear visualizations and timely alerts.

Also, it is important to assert privacy issues and make sure that effective security procedures are in place to safeguard private patient information obtained via non-invasive sensors. Maintaining patient trust and ensuring ethical use of the gathered data depend on compliance with data protection laws and regulations. Machine learning were used widely for the identification of the heart failure clinical cases, although accuracy is affected by algorithm type and data size, frame rates of the imaging outcomes and physician intervention is critical leading to different models from hospital to the other.

Deep learning approaches—which are more potent and effective ways to handle the enormous volumes of data generated—evolved from machine learning approaches, has proven to have potential for use in heart failure modeling. Although it is a black box technique, some studies showed promising results on evaluating the algorithms based on how clinical end point choice is made.

## 5 Conclusion

Heart dysfunction is the cause of worrisome patient outcomes, a subsequent high-rate mortality, and increased economic loss. The creation of precise and individualized models for heart failure prediction, diagnosis, and management has been made possible by the integration of non-invasive sensing technologies including BCG, ICG, PPG, ECG, and imaging-based heart simulators. These sensor-based models can offer insightful information about the development and severity of heart failure by using analytics and machine learning approaches. To enhance patient outcomes and quality of life, they have the capacity to identify early warning signals, predict exacerbations, and perfect treatment approaches. Furthermore, the potential for remote monitoring provided by non-invasive sensor-based technology allows healthcare personnel to track patient status from a distance and take proactive action when necessary. This can result in prompt interventions, fewer hospital stays, and better patient management, especially for those with chronic or severe heart failure.

The widespread application and adoption of non-invasive sensor-based technology for heart failure modeling still faces difficulties, nevertheless. Data integration, interoperability, privacy issues, and the requirement for validation across a range of patient populations are some of these difficulties. To guarantee that these technologies are useful and prevalent for patients and healthcare professionals, it is also crucial to address their usability and accessibility issues.

To sum up, non-invasive sensor-based technologies have enormous potential for developing heart failure, systolic and diastolic dysfunction modeling. These technologies have the potential to transform cardiac dysfunction management and greatly enhance patient care and outcomes with sustained study, technological breakthroughs, and collaboration between researchers, engineers, and physicians.
